# Treatment of Advanced Stage Gonarthrosis With Prolotherapy: Case Report

**DOI:** 10.5812/aapm.9171

**Published:** 2013-12-16

**Authors:** Ilker Solmaz, Suleyman Deniz, Onder Taylan Cifci

**Affiliations:** 1 Turkey Proloteraphy and Pain Clinic, Istanbul, Turkey; 2 Department of Anesthesiology, Gulhane Military Medical Academy, Haydarpasa Training Hospital, Istanbul, Turkey; 3 Gumussuyu Military Hospital, Istanbul, Turkey

**Keywords:** Osteoarthritis, Knee, Degeneration, Treatment

## Abstract

**Introduction:**

This case report aims to discuss the clinical and radiological outcomes of prolotherapy at a patient whom to total knee prosthesis had been planned but surgery couldn’t be performed due to co-morbidities.

**Case Presentation:**

A 72 year old woman presented with severe pain at her knees for over 5 years. Treatment methods include weight loss, decreasing the weight bearing on the joint, stretching exercises, nonsteroid anti-inflammatory and steroid drugs, and physiotherapy. The Western Ontario and McMaster Universities Osteoarthritis Index (WOMAC) scale was applied to measure the osteoarthritis level of the patient: Pain level; 25 points, stiffness level; 10 points, Physical function loss; 80 points, and total WOMAC 115 points. At radiological evaluation, the patient was diagnosed as grade IV osteoarthritis due to significant osteophyte presence and complete joint space narrowing. Six sessions of knee prolotherapy protocol was applied to the patient, one session monthly.

**Conclusions:**

Significant improvement was noted at WOMAC scale (Pain level; 5 points, stiffness level; 2 points, Physical function loss; 15 points, and total WOMAC 22 points). Osteoarthritis level of the patient was improved to grade I at radiological evaluation after a year. Our case is the report that presents radiological evidence in addition to clinical findings of improvement of osteoarthritis level. As a result of this case report, further studies aiming to offer a different minimally invasive treatment option to the patients before surgery may be performed. We are in the opinion that prolotherapy may be preferred more commonly as an efficient method once the importance of ligamentous structures at pathogenesis of osteoarthritis is established.

## 1. Introduction

Degenerative joint disease (osteoarthritis) is a chronic, non-inflammatory and common joint disease characterized with degeneration of synovial joint cartilage, and new bone formation at joint surfaces and margins (1).

Osteoarthritis is the most common joint disease and one of the most common causes of physical deformity. It affects both genders and all races. Although 30 % of individuals above 75 years old have symptoms, non-symptomatic (radiologic) osteoarthritis is presented over 20 % of the patients at 3rd decade and 80 % at 8th decade (2). 

Knee is the most commonly affected joint, particularly due to weight bearing. Knee osteoarthritis may significantly impact an individual’s quality of life; even make walking impossible (2).

Treatment methods include weight loss, decreasing the weight bearing on the joint, stretching exercises, nonsteroid anti-inflammatory and steroid drugs, and physiotherapy. On the other hand, a pathological process including exchange of joint surface with metals may be preferred at orthopedic approach (1, 2). 

Prolotherapy is an injection therapy method that is capable of reversing the degeneration process by activating the regeneration potential of the body. This is the only method capable of avoiding the patient from major surgical procedures by providing clinical improvement at even grade IV gonarthrosis (3).

This case report aims to discuss the clinical and radiological outcomes of prolotherapy at a patient whom to total knee prosthesis had been planned but surgery couldn’t be performed due to co-morbidities.

## 2. Case Presentation

A 72 year old woman presented with severe pain at her knees for over 5 years. She had history of chronic obstructive pulmonary disease for 10 years, diabetes mellitus for 20 years, and hypertension for 30 years. In addition to physiotherapy, intra-articular steroid two years ago and hyaluronic acid injections one year ago had been applied, and her complaints had not resolved with these treatments, although she used 500 mg paracetamol, 30 mg caffeine, 10 mg codein every six hour and 25 mg dexketoprofen trometamol every eight hour.

The last physician patient was referred had recommended total knee prosthesis but stated that surgical intervention would be unfavorable due to her irregular chronic diseases. After all, the patient was referred to our clinic. 

Physical examination revealed tenderness at medial and lateral collateral ligaments, pes anserius, patellar ligament, and coronary ligament. There was a decrease at joint flexion angle, range of motion (ROM = 90 degree) and stress tests were positive.

Visual Analog Scale (VAS), established by Price et al. (4), was used to measure the pain level of the patient. Pain level was detected as close to the most severe pain level (Scala 1).

The Western Ontario and McMaster Universities Osteoarthritis Index (WOMAC) scale (5) was applied to measure the osteoarthritis level of the patient: Pain level; 25 points, stiffness level; 10 points, Physical function loss; 80 points, and total WOMAC 115 points. 

**Figure 1.**
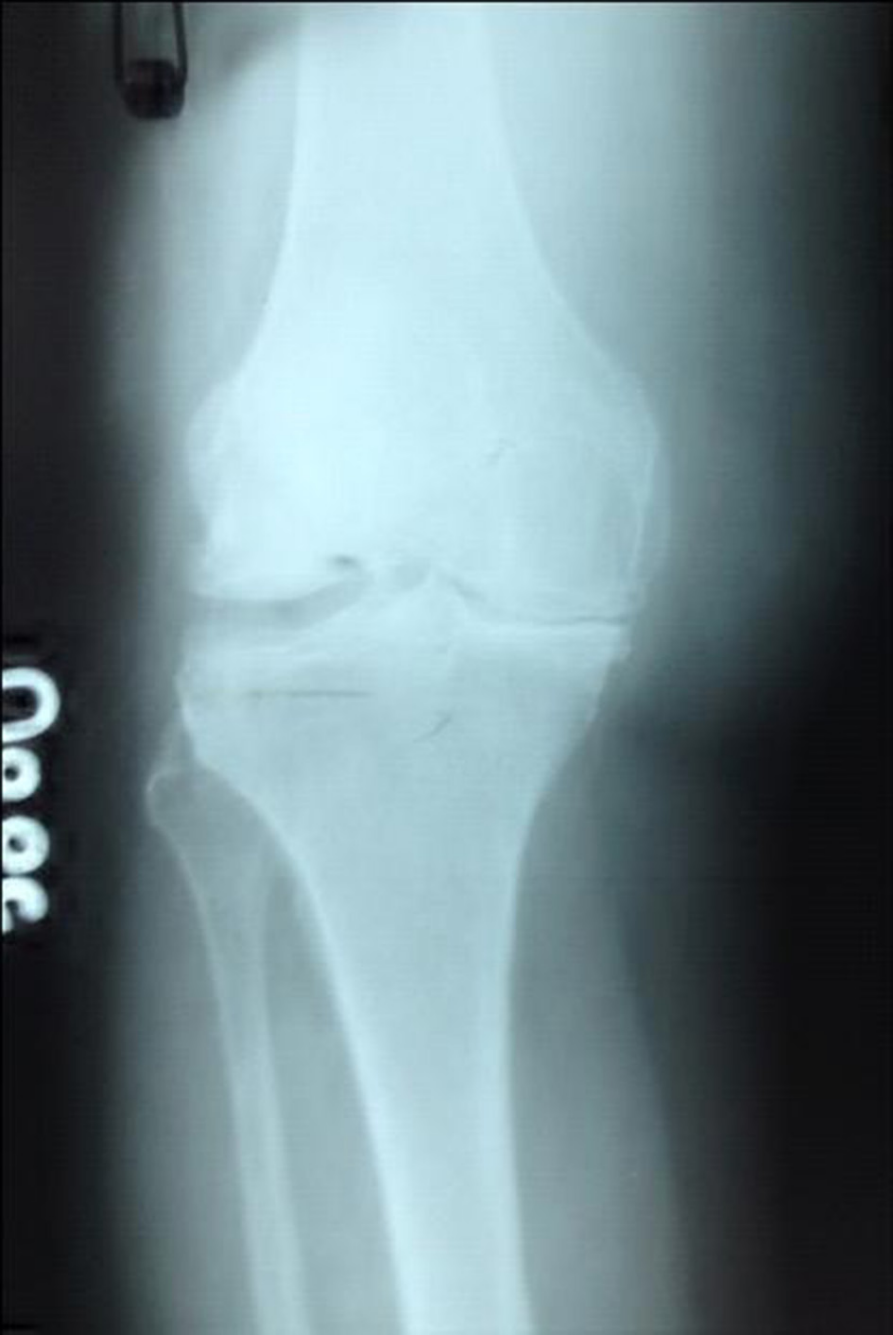
Knee Radiography Before Prolotherapy

At radiological evaluation, the patient was diagnosed as grade IV osteoarthritis due to significant osteophyte presence and complete joint space narrowing (Table 1) (Figure 1). 

**Table 1.** Radiological Knee Staging of Gonarthrosis Patients

Stage	Explanation
**1**	Minimal osteophyte, normal joint space
**2**	Significant osteophyte, suspicious joint space narrowing
**3**	Significant osteophyte and significant joint space narrowing
**4**	Significant osteophyte and complete joint space narrowing

Blood analysis and biochemical tests were reported as normal. Administration of prolotherapy protocol was decided after getting the written informed consent from the patient.

### 2.1. Therapy Protocol

Six sessions of knee prolotherapy protocol was applied to the patient, one session monthly.

The patient was monitored with ECG, arterial blood pressure and pulse oximeter measurements. 50 mcg fentanyl, 1 mg midazolam, and 50 mg propofol were given to the patient in divided doses for sedoanalgesia.

Tenderness points at examination were marked after sterilizing the injection area. 4 cc of 25% dextrose + 4 cc of 0.2% lidocaine solution was injected intra-articularly. Lateral knee injections.

0.5 cc of 15% dextrose + 0.5 cc of 0.2% lidocaine solution was injected to per certain points around the joint (joint capsule, insertion of medial coronary ligament, insertion of medial collateral ligament, teno-periosteal junction, insertion of pes anserious semimembranosus point over tibia, insertion of gastrocnemius and adductor magnus points over femur, fibro-osseous junction, insertion of arcuate and oblique ligaments, insertion of lateral coronary ligament, insertion of lateral collateral ligament, teno-periosteal junction, iliotibial tract at tibia, insertion of biceps femoris point at fibular head, insertion of gastrocnemius and popliteus points at femur). 

We administered five times the skin attempt for 15 point injections and used 22 cc solutions totally.

Nonsteroidal anti-inflammatory drugs and steroids have been lost. Weight loss, decreasing the weight bearing on the joint, stretching exercises and physiotherapy was continued.

## 3. Conclusions

The patient was evaluated at the end of 6 sessions. VAS score was measured as close to the no-pain point (Scala 2).

Significant improvement was noted at WOMAC scale (Pain level; 5 points, stiffness level; 2 points, Physical function loss; 15 points, and total WOMAC 22 points)

**Figure 2.**
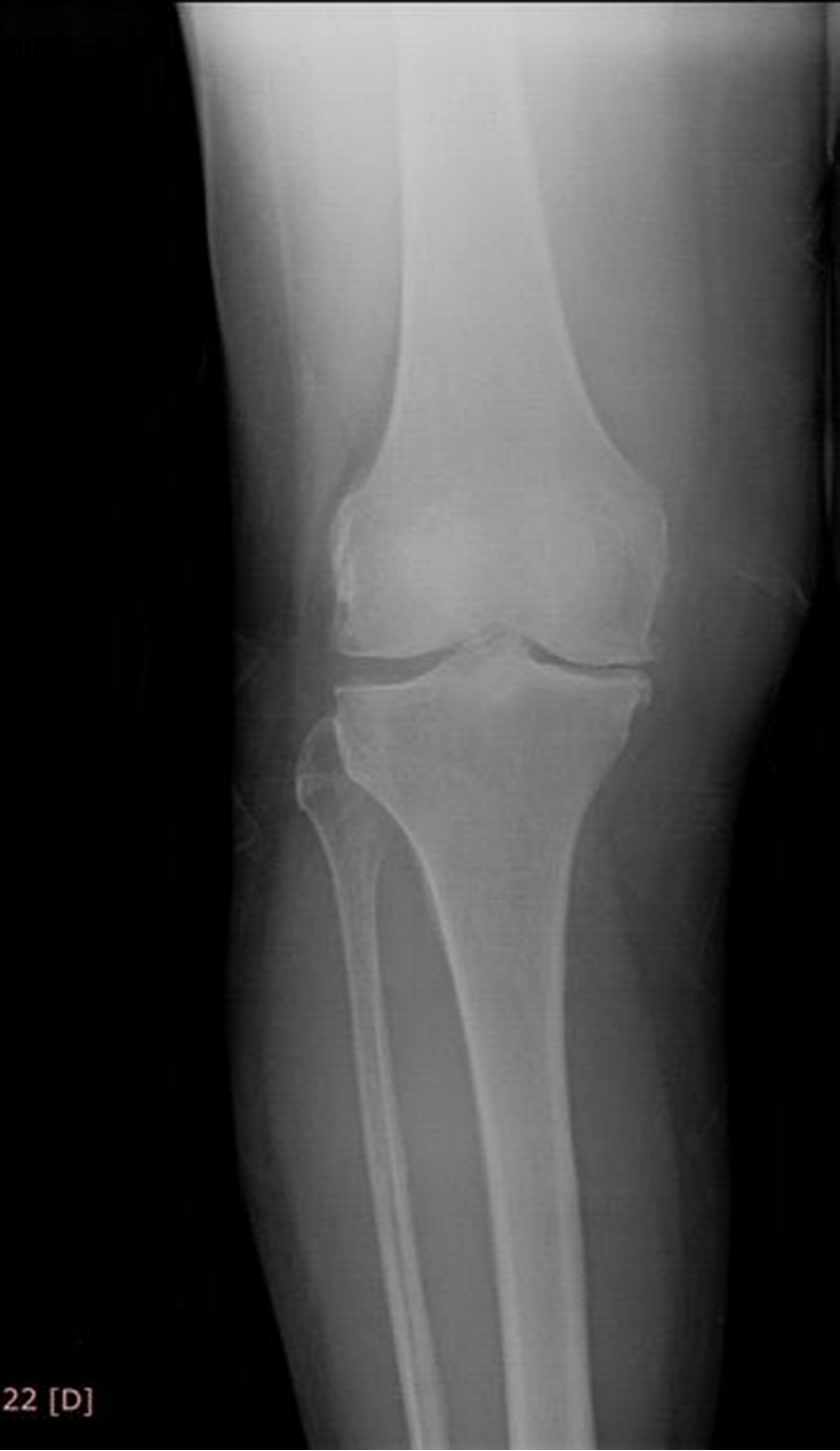
Knee Radiography After Prolotherapy

Osteoarthritis level of the patient was improved to grade I at radiological evaluation after a year (Figure 2). 

There was a increase at joint flexion angle, range of motion (ROM = 115 degree).

It is quite important that an improvement was shown at a patient with advanced stage gonarthrosis by clinical and radiological evidences after prolotherapy. In addition to the decrease at pain levels, grade IV arthritis was radiographically improved to grade I after a year. 

Injection therapies are commonly used for gonarthrosis as non-surgical treatment methods. The majority of these procedures include either corticosteroids or hyaluronic acid. These therapies decrease the complaints at short-term. But no evidence could be found that they improve arthritis level at long-term (1-3, 6, 7). Clinical and radiological improvements were demonstrated with our therapy method. 

A limited number of double-blinded, randomized studies are found in the literature. The study by Hackett et al. (6) in 1960 is the first review. The studies of Reeves et al. (8-10) are the other studies demonstrating evidence of significant clinic improvements at patients after prolotherapy. 

Prolotherapy is a procedure which proliferative solutions are injected into ligamentous structures for regeneration. Injected solution causes inflammation at connective tissue. The immune response to inflammation regenerates ligamentous compounds and resolves ligamentous laxity occurring at joint during arthritis (6, 7).

The reason of degeneration at synovial joint cartilage which has role at the pathology of osteoarthritis has not been well-established yet (6, 7). In our case, prolotherapy has been successful at reversal of the degeneration. Thus, ligamentous structures may be considered to have a key role at formation of arthritis. The stability of the joint is maintained by ligamentous structures. The impairment at joint mechanics due to laxity and degeneration at these structures may cause excessive weight bearing at synovial tissues. Cartilage degeneration, a reason for the complaints, may be the last step of the degeneration at pathological pathway; not the onset of the arthritis (7). 

The reason that therapies concerning only synovial cartilage provide only temporary improvements is these therapies don’t strengthen important ligamentous structures. The improvement at joint mechanics after regeneration of ligamentous structures may result with synovial regeneration (7). Ligamentous approach may offer new solutions at the treatment of arthritis and prevent plenty of patients from major surgical procedures.

Our case is the report that presents radiological evidence in addition to clinical findings of improvement of osteoarthritis level. 

As a result of this case report, further studies aiming to offer a different minimally invasive treatment option to the patients before surgery may be performed.

We are in the opinion that prolotherapy may be preferred more commonly as an efficient method once the importance of ligamentous structures at pathogenesis of osteoarthritis is established.
